# Constitutive Pleiotrophin Deletion Results in a Phenotype with an Altered Pancreatic Morphology and Function in Old Mice

**DOI:** 10.3390/ijms252010960

**Published:** 2024-10-11

**Authors:** Cristina Ballesteros-Pla, Julio Sevillano, María Gracia Sánchez-Alonso, María Limones, Jimena Pita, Begoña Zapatería, Marta Inmaculada Sanz-Cuadrado, Javier Pizarro-Delgado, Adriana Izquierdo-Lahuerta, Gema Medina-Gómez, Gonzalo Herradón, María del Pilar Ramos-Álvarez

**Affiliations:** 1 Departamento de Química y Bioquímica, Facultad de Farmacia, Universidad San Pablo-CEU, CEU Universities, Urbanización Montepríncipe, Boadilla del Monte, 28660 Madrid, Spain; cristina.ballesterospla@ceu.es (C.B.-P.); jsevilla@ceu.es (J.S.); maria.sanchezalonso@ceu.es (M.G.S.-A.); maria.limonescornejo@ceu.es (M.L.); jimena.pitasantibanez@ceu.es (J.P.); begona.zapateriagomez@einsteinmed.edu (B.Z.); m.sanz36@usp.ceu.es (M.I.S.-C.); javier.pizarrodelgado@ceu.es (J.P.-D.); 2 Department of Medicine, Marion Bessin Liver Research Center, Albert Einstein College of Medicine, Bronx, NY 10461, USA; 3 Departamento de Ciencias Básicas de la Salud, Universidad Rey Juan Carlos, Alcorcón, 28922 Madrid, Spain; adriana.izquierdo@urjc.es (A.I.-L.); gema.medina@urjc.es (G.M.-G.); 4 Departamento de Ciencias Farmacéuticas y de la Salud, Facultad de Farmacia, Universidad San Pablo-CEU, CEU Universities, Boadilla del Monte Urbanización Montepríncipe, 28660 Madrid, Spain; herradon@ceu.es

**Keywords:** glucagon, glucose transporter 2 (GLUT2), insulin, pancreatic islets, pleiotrophin, synaptosome-associated protein 25 (SNAP25), vesicle-associated membrane protein 2 (VAMP2)

## Abstract

Pleiotrophin (PTN) is crucial for embryonic development and pancreas organogenesis as it regulates metainflammation, metabolic homeostasis, thermogenesis, and glucose tolerance. Pleiotrophin deletion is associated with a lipodystrophic phenotype in which adipose tissue plasticity is altered in late life. This study explored the impact of pleiotrophin deletion on pancreatic morphology and function in later life. We analyzed glucose tolerance and circulating parameters on female wild-type (*Ptn*^+/+^) and knock-out (*Ptn*^−/−^) mice. At 9 and 15 months, we conducted morphometric analyses of pancreatic islets and evaluated the levels of insulin, glucagon, somatostatin, glucose transporter 2 (GLUT2), vesicle-associated membrane protein 2 (VAMP2), and synaptosome-associated protein 25 (SNAP25) via immunofluorescence. The effect of PTN on glucose-stimulated insulin secretion (GSIS) was evaluated in INS1E cells and isolated islets. *Ptn*^−/−^ mice showed hyperinsulinemia, impaired glucose tolerance, and increased homeostatic model assessment for insulin resistance (HOMA-IR) with age. While *Ptn*^+/+^ islets enlarge with age, in *Ptn*^−/−^ mice, the median size decreased, and insulin content increased. Vesicle transport and exocytosis proteins were significantly increased in 9-month-old *Ptn*^−/−^ islets. Islets from *Ptn*^−/−^ mice showed impaired GSIS and decreased cell membrane localization of GLUT2 whereas, PTN increased GSIS in INS1E cells. *Ptn* deletion accelerated age-related changes in the endocrine pancreas, affecting islet number and *size*, and altering VAMP2 and SNAP25 levels and GLUT2 localization leading to impaired GSIS and insulin accumulation in islets.

## 1. Introduction

Pleiotrophin (PTN) is a mitogenic cytokine widely expressed during embryonic development and in the brain during adulthood [1,2,3,4,5]. Additionally, PTN expression is upregulated in inflammatory processes [6] and during cellular proliferation and differentiation [7,8,9,10]. Although to date, the majority of its effects have been studied in the central nervous system and in cancer, PTN is implicated in the differentiation of preadipocytes, and it has been postulated as a key protein in the regulation of adipogenesis [11]. Moreover, PTN is involved in the regulation of neuroinflammation [6,12], metabolic homeostasis and metainflammation [13], and in adipose tissue browning [14]. In fact, PTN has been suggested to be a druggable target for metabolic disorders [15] as it is involved in the crosstalk between white and brown adipose tissue regulating thermogenesis [13,14], and in the liver accumulation of lipids in high-fat diet-induced obesity [13].

Research on the effects of pleiotrophin on the development of the pancreas, islet size and number, and insulin expression and/or secretion is scarce. Apart from the involvement of PTN in the increased perineural invasion in pancreatic cancer [16], preliminary previous studies have suggested a role in the pancreas organogenesis during development [17]. In fact, PTN is expressed in the pancreatic epithelium of mice at embryonic day (E) 11–13, and at E18 in blood vessels [18]. Pleiotrophin could also affect the development of insulin and glucagon endocrine precursor cells as well as exocrine differentiation [18], and intense PTN staining was observed during beta-cell regeneration in young mice after beta-cells depletion via streptozotocin [19,20]. Furthermore, *Ptn* expression is also maintained in the adult pancreas of several species such as mouse, rat, and human and increased in insulin-expressing but glucose-transporter-2-low (Ins + Glut2^LO^) progenitor cells [20]. Several receptors for PTN have previously been described (reviewed in [15,21]). In beta-cells, PTN has been proposed to exert its actions through integrin αvβ3 and receptor protein tyrosine phosphatase (RPTP) β/ζ [20,22].

As described in a recent study, exogenous administration of recombinant PTN induces the expression of pancreatic duodenal homebox 1 (*Pdx-1*) and insulin genes and favors beta-cell mass expansion, although preliminary studies with recombinant PTN administration do not seem to directly alter insulin secretion [20]. Nonetheless, some studies have pointed out that PTN could induce metainflammation [23], which would eventually lead to beta-cell dysfunction and consequently, alter insulin secretion.

To guarantee the maintenance of glucose homeostasis, the response of beta-cells is coordinated with that of alpha-cells which produce and secrete glucagon. Upon beta- and/or alpha-cell dysfunction or death, insulin and glucagon secretion are impaired which leads to hyperglycemia, the hallmark of diabetes [24]. In this situation, the main insulin-sensitive metabolic organs, such as the adipose tissue or the skeletal muscle, uptake glucose less efficiently, which leads to a whole status of insulin resistance [25], as with aging. Therefore, understanding the functional changes that beta-cell function undergoes with age can reveal new therapeutic targets and strategies to delay or reverse the disease.

Pleiotrophin deletion has been shown to be associated to a decreased body weight both in young and old *Ptn^−/−^* mice. However, *Ptn^−/−^* mice evolve from a higher degree of adiposity phenotype in young animals to a slimness in older mice. In fact, whereas at 3 months of age, adiposity was higher in *Ptn* knock-out mice when compared to their wild-type counterparts, at 15 months of age, the absence of *Ptn* triggers a switch in all these parameters, rendering mice with lower adiposity, impaired adipose tissue plasticity, and diminished glucose tolerance [14].

The aim of this study was to investigate whether the lipodystrophic phenotype associated with altered adipose tissue plasticity and impaired glucose homeostasis in late life [14] is also associated with altered pancreatic morphology and function, particularly in relation to insulin secretion.

To test this hypothesis, we have used a *Ptn^−/−^* mouse model and analyzed, for the first time, the effects of pleiotrophin deletion on the number and size distribution of islets in the pancreata of 9- and 15-month-old mice and how this might alter islet content of insulin and glucagon. Additionally, to shed light on the molecular mechanism, we analyzed the vesicular transport proteins (vesicle-associated membrane protein 2 (VAMP2) and synaptosome-associated protein 25 (SNAP25)), as well as the localization of GLUT2 glucose transporter and the basal and glucose-stimulated insulin secretion (GSIS).

## 2. Results

### 2.1. Pleiotrophin Deletion Is Associated with Age-Related Changes in Glucose–Insulin Homeostasis

**Figure 1:**
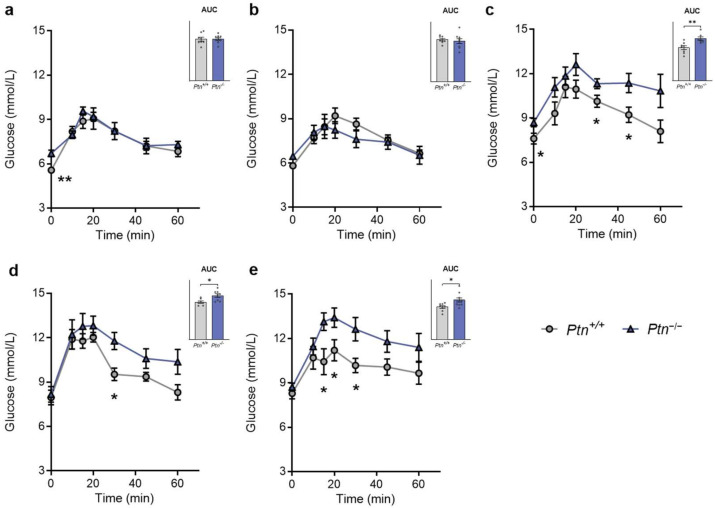
Glucose tolerance test (IPGTT) in *Ptn*^+/+^ (grey circles) and *Ptn*^−/−^ (blue triangles) mice. Changes in blood glucose concentration and areas under the glucose curve (AUC) in 6 h fasted mice in the glucose tolerance tests at: (**a**) 3 months, (**b**) 6 months, (**c**) 9 months, (**d**) 12 months, and (**e**) 15 months of age. Plasma glucose was measured at 0, 10, 15, 20, 30, 45, and 60 min after intraperitoneal administration of glucose (2 g/kg). Values are expressed as mean ± SEM of *n* = 8 mice/group. Differences between *Ptn*^−/−^ vs. *Ptn*^+/+^ mice are shown by: *: *p* < 0.05; **: *p* < 0.01.

To test whether the absence of PTN affected glucose tolerance during aging we performed intraperitoneal glucose tolerance tests (IPGTT) in 3-, 6-, 9-, 12- and 15-month-old *Ptn^−/−^* and *Ptn^+/+^* mice. At 3 and 6 months of age, mice from both genotypes presented similar curves for glucose (Figure 1a,b). However, in 9-, 12- and 15-month-old *Ptn^−/−^* mice, the AUC of glucose was higher than in *Ptn^+/+^* mice. Although the basal glycemia was similar in both 12- and 15-month-old *Ptn^−/−^* and *Ptn^+/+^*, there was an increment in the highest peak glucose value in 9-, 12- and 15-month-old *Ptn*-deficient mice. In fact, *Ptn^−/−^* mice also showed an impaired recovery of glycemia to basal glucose levels after 60 min when compared to their wild-type counterparts (Figure 1c–e). Altogether, these results suggest an impaired glucose tolerance in the *Ptn^−/−^* mice during later life.

**Figure 2:**
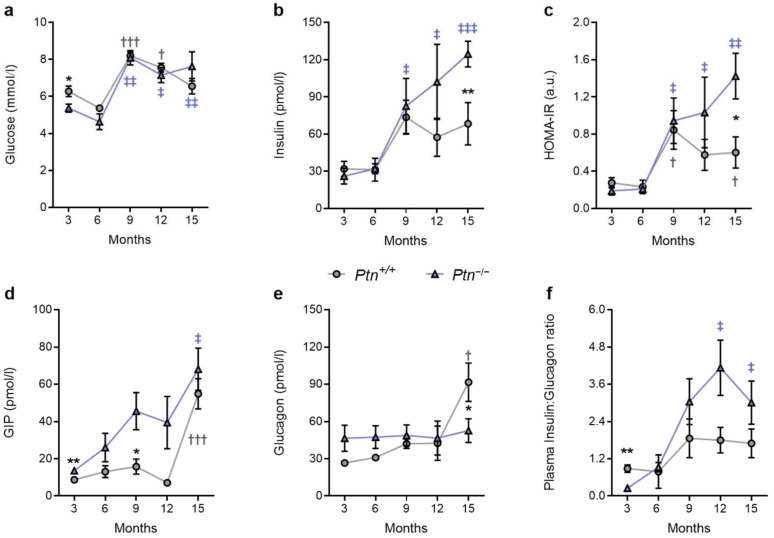
Plasma concentration of glucose, insulin, GIP, glucagon, and HOMA-insulin resistance index in *Ptn*^+/+^ (grey circles) and *Ptn*^−/−^ (blue triangles) mice at 3, 6, 12, and 15 months of age. Fasting (**a**) glucose (mmol/l), (**b**) insulin (pmol/l), (**c**) HOMA-IR, (**d**) GIP (gastric inhibitory polypeptide) (pmol/l), (**e**) glucagon (pmol/l) and (**f**) plasma insulin/glucagon ratio. Data are mean ± SEM of *n* = 8 mice/group. Statistical comparisons for the effect of aging within each group (vs. 3 months) are indicated as: ^†^ *p* < 0.05, ^†††^ *p* < 0.001 for *Ptn*^+/+^ mice; ^‡^ *p* < 0.05, ^‡‡^ *p* < 0.01, ^‡‡‡^ *p* < 0.001 for *Ptn*^−/−^ mice. Statistical comparisons for the effect of genotype are indicated as * *p* < 0.05; ** *p* < 0.01.

Fasting glycemia significantly increased at 9 months of age. Statistical analyses showed that differences regarding glucose were only due to age, and no influence of the genotype was found, except at 3 months age, being lower in *Ptn^−/−^* than in *Ptn^+/+^* mice (Figure 2a). Insulin levels were significantly higher in *Ptn*^−/−^ in later stages of life (15 months) when compared to wild-type animals (Figure 2b). To evaluate the influence of the genotype on insulin resistance, we calculated the HOMA-IR index for both *Ptn^−/−^* and *Ptn^+/+^* fasted mice (Figure 2c). Pleiotrophin deletion was associated with a higher HOMA-IR value at 15 months of age.

We next studied the impact of *Ptn* deficiency on circulating gastric inhibitory polypeptide (GIP) and glucagon. From 3 to 12 months of age, plasma concentration of GIP was significantly higher in the *Ptn^−/−^* mice when compared to wild-type animals (Figure 2d); however, at 15 months of age, GIP levels increased similarly regardless of the genotype. Although no significant differences were observed in circulating glucagon from 3 to 12 months between both genotypes, at 15 months of age there was a drastic increase in the concentration of this hormone in the *Ptn^+/+^* (Figure 2e) but not in the *Ptn*^−/−^ animals. The disproportionate changes in the plasma concentration of insulin and glucagon are evident when the insulin–glucagon (I/G) ratio was calculated. As shown in Figure 2f, although 3-month-old *Ptn*^−/−^ mice exhibited a lower I/G ratio, at 6 months of age no differences between genotypes were found. In addition, whereas in *Ptn^+/+^* mice no significant changes were observed during aging, a significant increase in the ratio was found in *Ptn*^−/−^ animals.

### 2.2. Ptn Deletion Alters Pancreas Islet Number and Size Distribution

**Figure 3:**
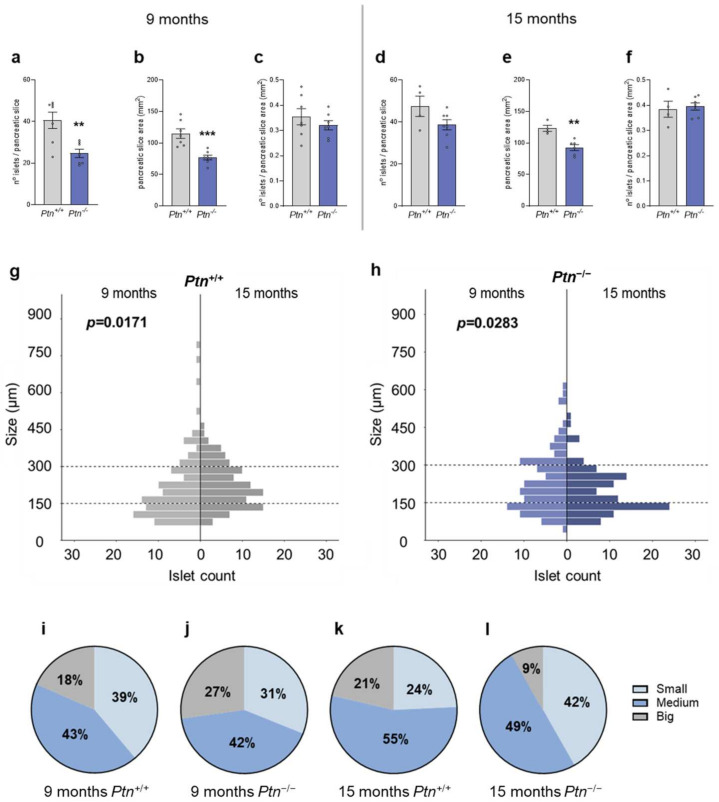
Pancreatic islets size and number distribution in 9- and 15-month-old *Ptn^+/+^* and *Ptn^−/−^* mice. Islet count per pancreatic slice (**a**), pancreatic slice area (mm^2^) (**b**), proportion of islets/pancreatic slice area (mm^2^) (**c**) at 9 months. Islet count per pancreatic slice (**d**), pancreatic slice area (mm^2^) (**e**), proportion of islets/pancreatic slice area (mm^2^) (**f**) at 15 months. Data are represented as mean ± SEM for *n =* 7 mice/group for 9 months *Ptn^+/+^* mice, *n =* 4 mice/group for 15 months *Ptn^+/+^* mice, *n =* 7 mice/group for 9 months *Ptn^−/−^* mice and *n =* 7 mice/group for 15 months *Ptn^−/−^* mice. Differences between *Ptn*^−/−^ vs. *Ptn*^+/+^ mice are shown by: ** *p* < 0.01, *** *p* < 0.001. Histograms representing size (µm)-number distribution of 103 islets counted in (**g**) *Ptn^+/+^* at 9 months vs. 15 months of age and (**h**) in *Ptn^−/−^* at 9 months vs. 15 months of age. Differences in distribution are represented as Mann–Whitney U test *p* values. Pie charts are used to represent percentages of small (<150 µm), medium (150–300 µm), and big (>300 µm) pancreatic islets in the following age groups: (**i**) 9 months *Ptn^+/+^* mice, (**j**) 9 months *Ptn^−/−^* mice, (**k**) 15 months *Ptn^+/+^* mice and (**l**) 15 months *Ptn^−/−^* mice. Dashed lines in figures (**g**) and (**h**) represent cut off for small, medium and big islets.

*Ptn*^−/−^ mice showed a decreased pancreas weight both at 9 months (0.2660 ± 0.0183 vs. 0.1650 ± 0.0141 g for *Ptn^+/+^* and *Ptn*^−/−^ mice, respectively, *p* = 0.0242) and at 15 months of age (0.2319 ± 0.0145 vs. 0.18430 ± 0.0092 g for *Ptn^+/+^* and *Ptn*^−/−^ mice, respectively, *p* = 0.0289). Moreover, we determined in the hematoxylin–eosin-stained pancreatic slices, the number of islets and the area in each pancreatic slice and observed that, at 9 months of age, both parameters were significantly lower in the *Ptn*^−/−^ than in *Ptn^+/+^* mice (Figure 3a,b), although the proportion of islets per pancreatic slice area was similar in both genotypes (Figure 3c). At 15 months of age, *Ptn*^−/−^ mice showed a decreased average pancreas slice area (Figure 3e), although no differences were observed regarding islet count nor proportion of islets per pancreas slice area when compared to *Ptn^+/+^* mice (Figure 3d–f). Subsequently, we measured the major diameter of islets of 9- and 15-month-old mice of both genotypes and evaluated if age affected the distribution of size of the islets, and if *Ptn* deletion may have had an impact on this adaptation. For that, we analyzed the same number of Langerhans islets in each experimental group (103 islets) and ordered the data by the diameter of the islets. In *Ptn^+/+^* animals (Figure 3g), the distribution of size varied significantly with age and the median islets size increased in later stages of life (177.69 µm at 9 months and 211.30 µm at 15 months). The opposite effect was observed in *Ptn*-deficient mice (Figure 3h), as the median islets size decreased significantly with age (205.28 µm at 9 months and 171.35 µm at 15 months).

Additionally, we classified pancreatic islets according to major diameter into small, medium, and big islets. As shown in Figure 3i,k, the proportion of small islets was significantly diminished (*p* = 0.0245 Chi-square, 9 vs. 15 months) with age in wild-type animals. However, the percentage of small islets increased, and the number of big islets drastically diminished in the *Ptn^−/−^* mice with age (*p* = 0.0006 Chi-square, 9 vs. 15 months age) (Figure 3j,l). *Ptn* deletion did not significantly affect the proportion of small, medium, and big islets at 9 months of age, (Figure 3i,j) when compared to *Ptn^+/+^* mice. However, at 15 months of age, the proportion of small islets was higher (*p* = 0.0077 Chi-square) and the proportion of big islets smaller (*p* = 0.0113 Chi-square) in the *Ptn*^−/−^ mice relative to wild-type animals (Figure 3k,l). The comparisons of islets size proportions of all experimental groups are further detailed in Supplementary Tables S1 and S2.

### 2.3. Ptn Deletion Increases Beta-Cell-Insulin, Delta-Cell Somatostatin Content Alpha-Cell Number, and the Expression of Vesicular Transport-Proteins at 9 Months of Age

**Figure 4:**
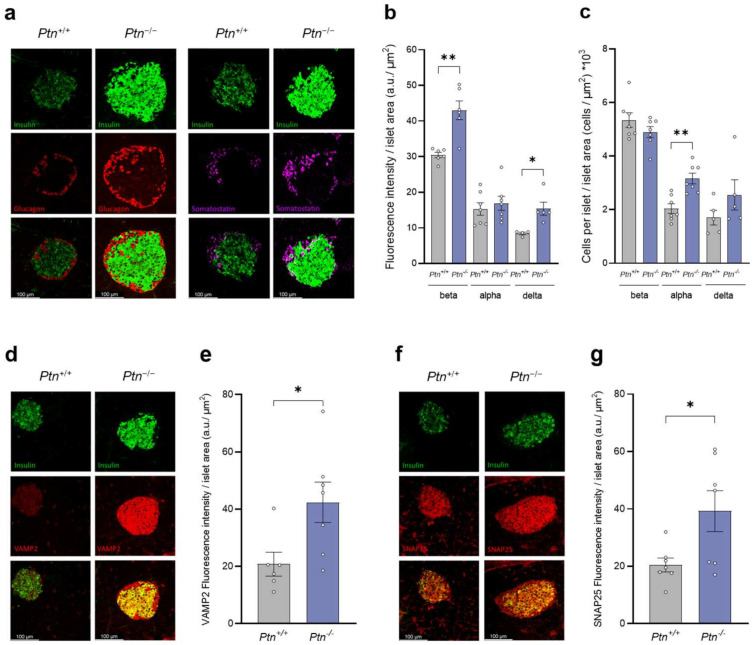
Immunofluorescence analysis of insulin, glucagon, somatostatin, and the transport-proteins VAMP2 and SNAP25 in *Ptn^+/+^* and *Ptn^−/−^* 9-month-old mice. (**a**) Z-stack of representative confocal images of a pancreatic section showing an islet immunostained for insulin (green, top panels), glucagon (red, first two mid left panels), and somatostatin (magenta, second two mid right panels). Bottom left and right panels represent the merged images. Scale bars: 100 µm. (**b**) Quantification of fluorescence intensity for insulin, glucagon, and somatostatin immunopositive cells in each islet relative to the corresponding islet surface area. (**c**) Quantification of beta-, alpha- and delta-cells number in each pancreatic islet relative to the corresponding surface area. (**d**) Z-stack of representative confocal images of a pancreatic section showing an islet immunostained for insulin (green, top left and right panels) and VAMP2 (red, first mid left and right panels). Bottom left and right panels represent the merged images. Scale bars: 100 µm. (**e**) Quantification of fluorescence intensity for VAMP2 immunopositive cells in each islet relative to the corresponding islet surface area. (**f**) Z-stack of representative confocal images of a pancreatic section showing an islet immunostained for insulin (green, top left and right panels) and SNAP25 (red, first mid left and right panels). Bottom left and right panels represent the merged images. Scale bars: 100 µm. (**g**) Quantification of fluorescence intensity for SNAP25 immunopositive cells in each islet relative to the corresponding islet surface area. Data are represented as mean ± SEM for *n* = 5–7 mice/group. Differences between *Ptn*^−/−^ versus *Ptn*^+/+^ mice are shown by: **: *p* < 0.01; *: *p* < 0.05.

Although immunofluorescence analysis of pancreas of 9-month-old mice revealed an increment in the insulin and somatostatin immunoreactivity–islet area in the *Ptn*^−/−^ compared to *Ptn^+/+^* mice, no differences were observed between genotypes in the glucagon immunoreactivity–islet area (Figure 4a,b), nor in the insulin–glucagon ratio (Supplementary Figure S1a). Moreover, although no differences were observed in the content of beta-cells nor delta-cells per islet area at 9 months of age, *Ptn*^−/−^ mice showed a higher proportion of alpha-cells per islet area than age-matched *Ptn^+/+^* mice (Figure 4c).

As all the immunofluorescence analyses were run in parallel and with a blind observer, these results suggest that the *Ptn* deletion renders mice with a higher insulin accumulation in the islets. Thus, we evaluated whether *Ptn^−/−^* mice may present any alteration in the mechanisms of vesicle transport. To achieve this, we explored the presence of VAMP2 (localized on the surface of the vesicles) and SNAP25 (localized in the cell membrane) proteins. As observed in Figure 4d–g, the immunofluorescence–islet area of both VAMP2 and SNAP25 was higher in the islets of 9-month-old *Ptn^−/−^* mice than in *Ptn^+/+^* mice.

### 2.4. The Absence of Ptn Affects Alpha–Beta Cell Proportion in the Islets of Langerhans in 15-Month-Old Mice

**Figure 5:**
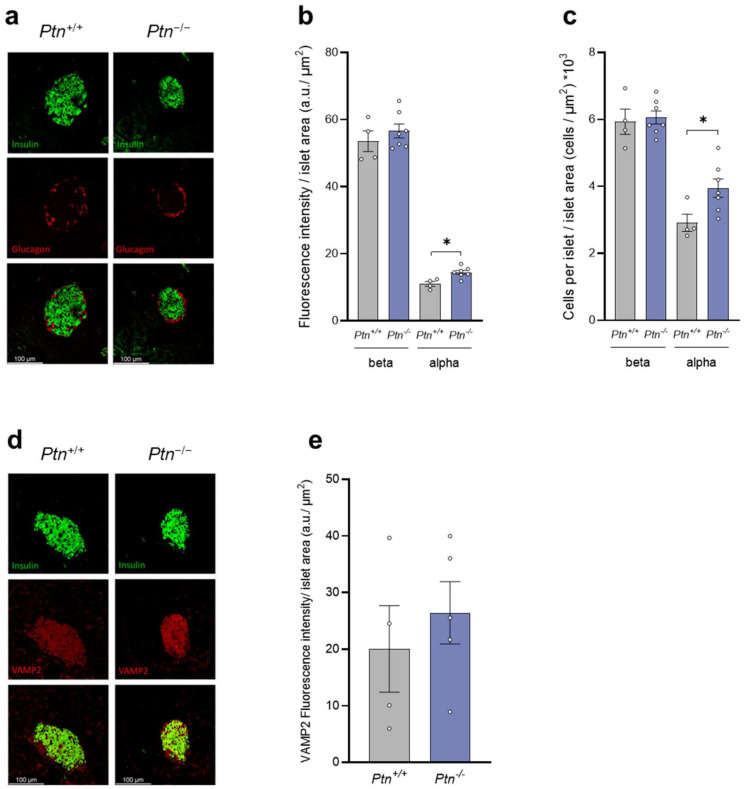
Immunofluorescence analysis of insulin, glucagon, and the transport-protein VAMP2 in *Ptn^+/+^* and *Ptn^−/−^* 15-month-old mice. (**a**) Z-stack of representative confocal images of a pancreatic section showing an islet immunostained for insulin (green, top left and right panels) and glucagon (red, first mid left and right panels). Bottom left and right panels represent the merged images. Scale bars: 100 µm. (**b**) Quantification of fluorescence intensity for insulin and glucagon immunopositive cells in each islet relative to the corresponding islet surface area. (**c**) Quantification of beta- and alpha-cells number in each pancreatic islet relative to the corresponding surface area. (**d**) Z-stack of representative confocal images of a pancreatic section showing an islet immunostained for insulin (green, top left and right panels) and VAMP2 (red, first mid left and right panels). Bottom left and right panels represent the merged images. Scale bars: 100 µm. (**e**) Quantification of fluorescence intensity for VAMP2 immunopositive cells in each islet relative to the corresponding islet surface area. Data are represented as mean ± SEM for *n =* 4 mice/group for *Ptn^+/+^* mice (insulin, glucagon and VAMP2), *n =* 7 mice/group for insulin and glucagon determination and *n* = 5 mice/group for VAMP2 determination for *Ptn^−/−^* mice. Differences between *Ptn*^−/−^ versus *Ptn*^+/+^ mice are shown by: *: *p* < 0.05.

We next analyzed the content of insulin, glucagon, and the vesicular transport-proteins in the pancreas of 15-month-old mice (Figure 5). Although we observed no differences in the fluorescent intensity–islet area for insulin-immunopositive cells between genotypes, the fluorescence intensity of glucagon–islet area was significantly increased in *Ptn^−/−^* when compared to *Ptn^+/+^* mice (Figure 5a,b). Accordingly, although the insulin–glucagon ratio (Supplementary Figure S1b) and the proportion of beta-cells per islet area were similar in both genotypes, alpha-cell population was significantly increased in *Ptn*^−/−^ when compared to *Ptn*^+/+^ mice (Figure 5c). Finally, no changes were observed between genotypes in VAMP2 (Figure 5d,e).

### 2.5. Ptn Deletion Reduces Glucose-Stimulated Insulin Secretion of Pancreatic Islets from 9- and 15-Month-Old Mice

**Figure 6:**
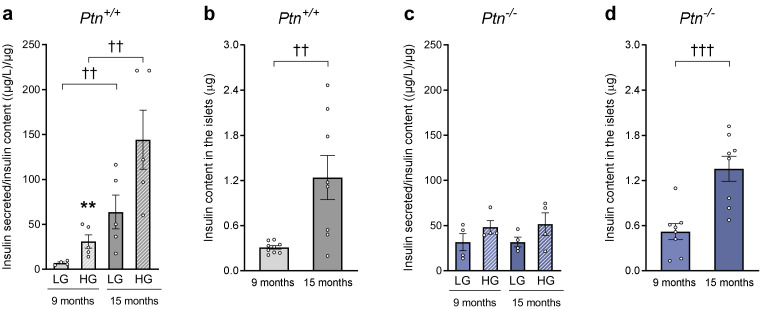
Glucose-stimulated insulin secretion (GSIS) and insulin content of 10 islets of 9- and 15-month-old *Ptn*^+/+^ and *Ptn*^−/−^ mice in the presence of low glucose (2.5 mM) and high glucose (25 mM). Data are represented as mean ± SEM for *n =* 5 mice/group for *Ptn^+/+^* mice, and *n =* 4 mice/group for *Ptn^−/−^* for GSIS (**a**,**c**). Data of insulin content of the islets were represented for each experimental group; no differences were observed regardless of incubation with either low or high glucose concentrations and represented as mean ± SEM for *n =* 9 mice/group for *Ptn^+/+^* mice, and *n =* 8 mice/group for *Ptn^−/−^* (**b**,**d**). Differences between low glucose and high glucose stimulation are shown by: **: *p* < 0.01. Differences between 9 months and 15 months are shown by: ^††^: *p* < 0.01; ^†††^: *p* < 0.001.

We further investigated whether aging or *Ptn* deletion could alter basal (2.5 mM glucose-low, LG) or glucose-stimulated (25 mM glucose-high, HG) insulin secretion from isolated islets. Basal insulin secretion from isolated islets (LG) significantly increased with age from 9- and 15-month-old *Ptn^+/+^* animals (Figure 6a) whereas no change was observed in *Ptn*^−/−^ animals (Figure 6c). Interestingly, isolated islets from 9-month-old *Ptn*^−/−^ mice exhibited a higher basal insulin secretion normalized via total islet insulin content (31.55 ± 9.52 (µg/L)/µg) when compared to *Ptn*^+/+^ animals (7.01 ± 1.13 (µg/L)/µg), *p =* 0.01). In the wild-type mice, the isolated islets from 9-month-old mice showed a significant increase in glucose-stimulated insulin release (4.41-fold). However, the effect did not reach statistical significance (*p* = 0.0648) at 15 months of age. Surprisingly, as seen in Figure 6c, islets from both 9- and 15-month-old *Ptn*^−/−^ mice showed a clear reduction in the glucose-stimulated insulin release (increment from 2.5 to 25 mM glucose), when compared to wild-type (1.53- and 4.41-fold in *Ptn^−/−^* and *Ptn^+/+^*, respectively, at 9 months; 1.62- and 2.26-fold in *Ptn*^−/−^ and *Ptn*^+/+^, respectively, at 15-months age). The fold change of insulin secretion from low to high glucose is presented in Supplementary Table S3. Moreover, a significant increase was observed regarding the insulin content of the islets in 15-month-old mice when compared to 9-month-old mice in both *Ptn^+/+^* and *Ptn^−/−^* mice (Figure 6b,d).

### 2.6. Ptn Deletion Reduces Membrane GLUT2 in the Pancreatic Islets of 9- and 15-Month-Old Mice

**Figure 7:**
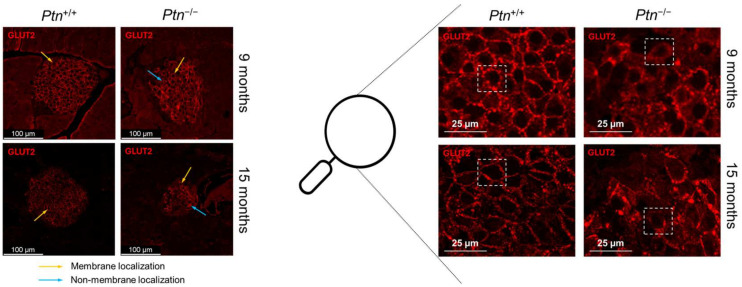
GLUT2 expression in 9- and 15-month-old female *Ptn^+/+^* and *Ptn^−/−^* mice. *Ptn^+/+^* mice show a more localized expression of GLUT2 in the membrane of islet cells (mainly beta-cells) both at 9 and 15 months when compared to *Ptn^−/−^* mice. White boxes in the augmented image (right part of the figure) indicate the most common pattern of GLUT2 membrane expression in islet cells for each animal group.

Considering the attenuated glucose-stimulated insulin secretion exhibited by isolated islets in *Ptn^−/−^* animals, we decided to analyze GLUT2, the principal glucose transporter within pancreatic islets. Immunofluorescence analyses revealed a differential distribution in *Ptn^+/+^* when compared to *Ptn^−/−^* mice. As depicted in Figure 7, mice lacking *Ptn* showed a reduction in GLUT2 in the membrane of beta-cells in comparison to their wild-type counterparts, at both 9 and 15 months of age. Furthermore, when we compare animals of the same genetic background but different ages, a decrease in the immunofluorescence of GLUT2 became apparent with advancing age, suggesting a decline in the amount of the protein. Consequently, we can attribute fluctuations in GLUT2 protein expression to a dual influence: genotype and age.

### 2.7. PTN Potentiates Glucose-Stimulated Insulin Secretion in INS1E

**Figure 8:**
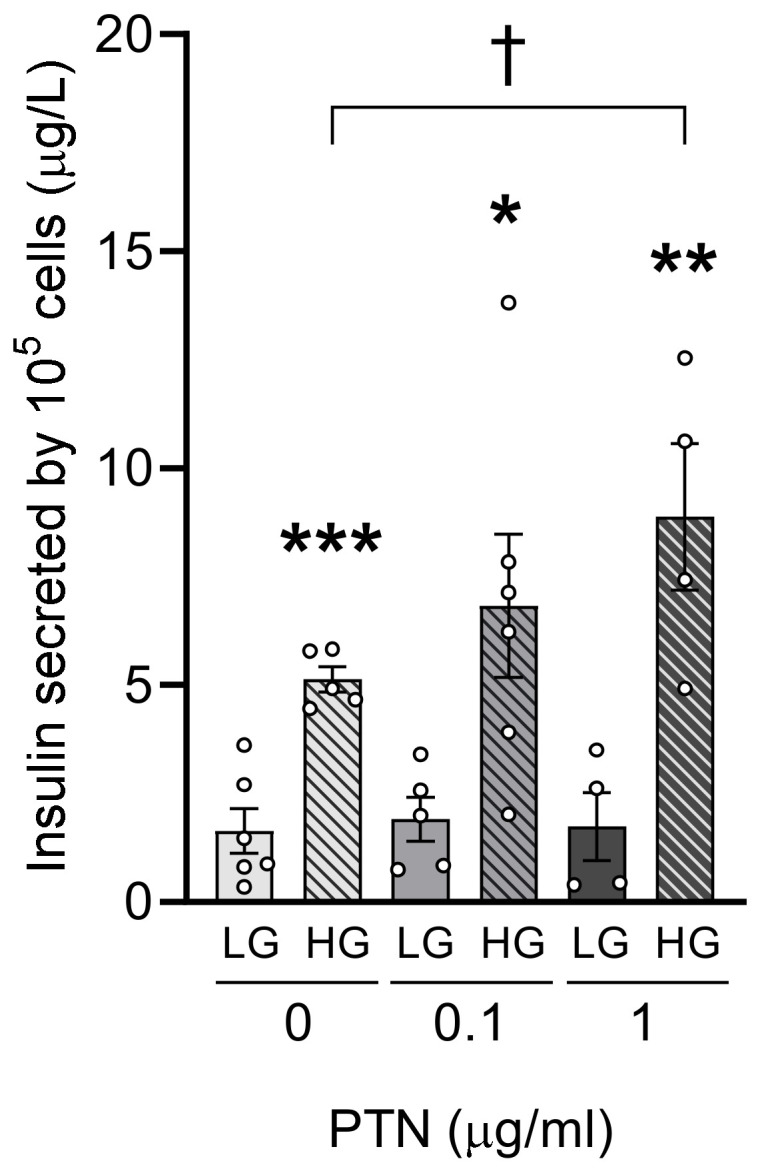
Insulin secretion (µg/L) by INS1E cells treated for 60 min with different concentrations of glucose (2 mM and 20 mM) and rPTN (0, 0.1, and 1 µg/mL). Solid bars indicate low glucose concentration (LG, 2 mM), while striped bars represent high glucose concentration (HG, 20 mM). Results are presented as mean ± SEM. Differences between low glucose and high glucose stimulation are shown by: *: *p* < 0.05; **: *p* < 0.01 ***: *p* < 0.001. Differences between concentrations of rPTN (versus 0 µg/mL) are shown by: ^†^: *p* < 0.05.

To further evaluate the direct effect of pleiotrophin on insulin secretion, we performed in vitro experiments to analyze the glucose-stimulated insulin secretion by the beta cell line INS1E in the presence and absence of recombinant pleiotrophin (rPTN). In all conditions, insulin secreted to the media was higher in the presence of high glucose (20 mM) than low glucose (2 mM); this effect was higher in the presence of rPTN (Figure 8). Spearman’s correlation analysis (r = 0.5707, *p* < 0.05) revealed a positive correlation between PTN concentration and the amount of insulin secreted under high glucose conditions (20 mM), indicating a dose–response effect. In fact, glucose-stimulated insulin secretion after 60 min was increased 3.1-fold for 0 µg/mL PTN, 3.6-fold for 0.1 µg/mL PTN, and 5.1-fold for 1 µg/mL PTN, being statistically significant for 1 µg/mL PTN.

## 3. Discussion

A previous study from our group pointed out the role of PTN, a growth factor that has also been involved in the embryonic development of endocrine and exocrine pancreas [18,20], in the regulation of glucose homeostasis with aging in females [14], leading us to hypothesize that constitutive *Ptn* deletion could alter pancreatic morphology and function, particularly in relation to insulin secretion at late life. Therefore, our objective was to investigate the role of PTN in the morphology and functionality of pancreatic islets, providing new evidence of a new target involved in the pathophysiology of metabolic disorders associated with glucose homeostasis.

Although glycemia increased with age similarly in both genotypes, the deletion of *Ptn* accelerates the development of glucose intolerance that occurs in aging. Thus, although young 3- and 6-month-old *Ptn^−/−^* mice exhibited an adequate glucose tolerance, as previously described [14], they became glucose intolerant at 9 months, and this effect persisted at 12 and 15 months of age.

Glycemia depends on metabolic control via the two main pancreatic hormones, insulin and glucagon. On one hand, aging induced glucose intolerance, hyperinsulinemia, and insulin resistance in both genotypes, but this increment was more pronounced in *Ptn^−/−^* mice. On the other hand, although aging induced a progressive increment in circulating glucagon in wild-type, levels were unchanged in *Ptn^−/−^* animals. This disproportionate change in the plasma concentration of the two hormones is more evident when the I/G ratio is considered, indicating an altered response in the aged *Ptn^−/−^* mice that it is not observed in wild-type animals. The I/G ratio changes with the need for anabolism and catabolism. In fact, the I/G ratio varies inversely with the requirement for endogenous glucose production, being lowest in starvation and highest during loading with exogenous carbohydrate. In a previous study [14], we demonstrated that *Ptn*-deficient mice use fatty acids as the main source of energy and to a lesser extent available glucose, even in a postpandrial state [14]. This adaptation results in a state of “glucose overload after food intake” that may explain the altered insulin–glucagon axis and the glucose intolerance of these animals.

Given the pronounced differences observed in *Ptn^−/−^* mice in glucose–insulin homeostasis from 9 months onwards, our primary focus was the characterization of the pancreas at 9 months of age, and then to evaluate progressive changes with aging (15 months), to analyze significant changes in pancreatic islet morphology and function in the elderly.

Firstly, our findings indicate that although the proportion of pancreatic islets per area was constant, PTN deficiency results in both a significant reduction in the number of islets and in the average pancreatic area when compared to wild-type animals at 9 and 15 months. These results emphasize the crucial role of PTN in pancreas morphology. In fact, the pancreata of *Ptn^−/−^* mice were significantly smaller than those of control animals. As PTN was involved in the development of endocrine and exocrine pancreas [18,20] during embryonic development, we cannot discard that the reduction observed in the islets in the *Ptn*-deficient mice may be a consequence of altered organogenesis, although an adaptive response to the glucose intolerance of these animals may also be involved.

Aging has been associated with an increase in the pancreatic islet area and size, without changes in the islet number [26]. Thus, we tested if the number and size of pancreatic islets were affected with age in our mouse model and found that aging produced significant changes in the number–size distribution of pancreatic islets in both genotypes in a different manner. On the one hand, wild-type animals displayed islet hypertrophy with age, as described before [26], which ultimately renders less functional islets in terms of hormone production and secretion. On the other hand, *Ptn^−/−^* mice exhibited a progressive decrease in islet size. Although controversial, in general terms, smaller islets have been described to be more functional regarding insulin production and secretion [27,28,29]. However, unstoppable progressive size reduction may also indicate a deterioration in pancreatic cell function and the onset of apoptosis, ultimately leading to insulinopenia, a characteristic feature of the advanced stages of type 2 diabetes [30]. These findings suggest that PTN plays a crucial role in preserving the structural integrity and functionality of pancreatic islets throughout the aging process.

Furthermore, insulin, glucagon, and somatostatin immunoreactivity in the pancreas of *Ptn^−/−^* mice revealed an increase in the insulin and somatostatin signal in the proportion of alpha-cells and a disrupted communication between beta-, alpha- and delta-cells in these animals. As it is known that somatostatin and insulin may inhibit glucagon secretion [31], the increased signals of both hormones in the pancreas from mice lacking *Ptn* may suggest an inhibition in glucagon secretion and consequently may account for the increase in alpha-cells as a compensatory mechanism. Previous studies have described an increased ratio of alpha-cells to beta-cells in the pancreatic islet of patients with type 2 diabetes [32,33]. This increase in the alpha–beta cell ratio may be due to either an increase in apoptotic processes leading to beta-cell loss, to dedifferentiation, or to transdifferentiation [34] processes. The beta-cell can transdifferentiate and become another cell type, normally an alpha-cell, although there can also be transdifferentiation to a delta-cell. These transdifferentiated cells may continue to express some of the markers of the original cell along with other markers of the new cell type. In addition, in some cases they may be bihormonal cells with the capacity to simultaneously produce insulin and glucagon [34]. Ongoing studies in our group are focused on elucidating the molecular mechanism underlying the altered ratio of alpha-cells to beta-cells in *Ptn^−/−^* mice.

To confirm if *Ptn* deletion may also affect beta-cell insulin secretion, we analyzed basal and glucose-stimulated insulin secretion in isolated islets from both genotypes. First, we found that islets from 15-month-old *Ptn*^+/+^ animals had an increased basal insulin secretion when compared to 9-month-old animals. This result was related to common alterations in insulin secretion mechanism due to aging [35,36]. However, no changes were observed in the pleiotrophin-deficient mice. Secondly, when we analyzed the effect of aging and genotype in glucose-stimulated insulin release, we found that the glucose-stimulated insulin secretion was completely blocked in *Ptn^−/−^* animals, whereas in the wild-type animals, isolated islets showed an increased insulin release in the presence of high glucose, being higher at 9 than at 15 months of age. As additional evidence of the direct participation of pleiotrophin in the glucose insulin-stimulated secretion (GSIS), we performed an in vitro study with INS1E cells finding that PTN significantly increased GSIS, which would support the effect observed in the isolated islet experiments from *Ptn*^+/+^ and *Ptn^−/−^* mice. Moreover, we also observed an increase in the total insulin content with age, however, there were very heterogeneous results, and no clear correlation has yet been found between insulin content and age [36]. The impaired alpha–beta crosstalk, together with the impaired insulin secretion in response to glucose, may favor the diabetogenic phenotype observed in *Ptn^−/−^* mice later in life.

To further investigate the molecular mechanisms that might be involved in the lack of glucose-stimulated insulin secretion of the islets of *Ptn^−/−^* mice, we analyzed GLUT2, as this transporter, which when localized in the cell membrane, modulates the glucose uptake by beta-cells. In fact, a previous in vitro study from our group revealed that incubation of INS1E rat beta-cells with PTN also increased the expression of *Glut-2* [20]. In our mouse model, a similar overall protein amount of GLUT2 was found in both genotypes. However, we observed a clear effect of both aging and genotype on GLUT2 cellular distribution. Indeed, in both genotypes, aging was associated with a decreased GLUT2 in the beta-cells, which was in consonance with what has been previously described by other groups [37,38,39]. Additionally, *Ptn* deletion reduced the amount of GLUT2 located in the cell membrane both at 9 and 15 months, which may compromise the transport of glucose inside the beta-cell in this genotype.

After beta-cell uptakes glucose through GLUT2, glucose oxidation causes an increase in the cytoplasmatic ATP–ADP ratio, which induces the closure of the K^+^-ATP channel and leads to the opening of the Ca^2+^ channel. Consequently, Ca^2+^ enters the beta-cells and promotes the migration of insulin-containing vesicles towards the cellular membrane, and the concomitant insulin secretion via exocytosis [40]. Since beta-cells from *Ptn^−/−^* animals have a decreased amount of GLUT2 in the cell membrane, glucose uptake capacity is reduced, and vesicles and insulin may accumulate within the pancreatic islet (Figure 8). In support of this, VAMP2 and SNAP25, vesicular transport-proteins that belong to the SNARE family were increased in islets of 9-month-old *Ptn^−/−^* mice. This increment in both insulin signal and vesicular transport-proteins in knock-out mice could be due to the accumulation of preformed vesicles within the islet, or as a result of a compensatory mechanism increasing insulin and vesicular transport-proteins to enhance insulin secretion and counteract glucose intolerance. However, at advanced ages (15 months), both insulin content and vesicular transport-proteins were similar in both genotypes. This lack of differences between genotypes at older ages might be a consequence of the exhaustion and consequently a progressive deterioration of the pancreatic beta-cell function with age in wild-type mice or to the effect of other secretagogues rather than glucose on insulin release in *Ptn*^−/−^ animals.

**Figure 9:**
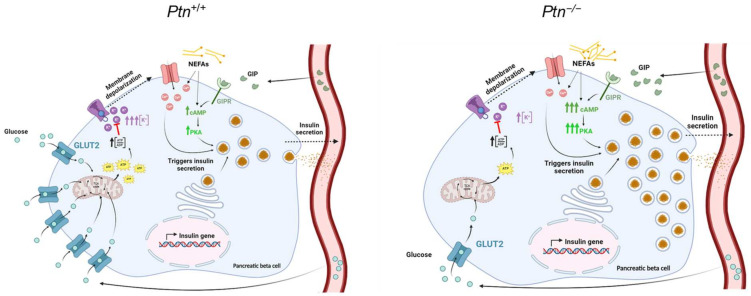
Graphical representation summarizing the main findings of the study by comparing the mechanism of insulin secretion in the pancreatic beta-cell of *Ptn*^−/−^ vs. *Ptn*^+/+^ animals. GIP (glucose-dependent insulinotropic polypeptide), GIPR (glucose-dependent insulinotropic polypeptide receptor), and PKA (protein kinase A). Red line indicates the inhibition of a process.

In this line of evidence and, taking into consideration the hyperinsulinemia of 15-months fasting *Ptn^−/−^* animals together with the decreased GLUT2 in the membrane of beta-cells, we speculate that insulin secretion in these pleiotrophin-deficient animals is mainly triggered by alternative mechanisms, other than glucose stimulation. Accordingly, other secretagogues, such as GIP, which is particularly elevated in knock-out animals between 3 and 12 months of age, may contribute to insulin secretion. In fact, the binding of GIP to its receptor rises intracellular cAMP that through activation of protein kinase A triggers the release of insulin vesicles to the bloodstream [41,42]. Furthermore, NEFA have shown to cause a rise in cytosolic Ca^2+^ [43], favoring not only glucose-independent insulin secretion [44], but also potentiating glucose-stimulated insulin secretion in an attempt to compensate insulin resistance [45] (Figure 9). Interestingly, previous studies of our group have shown a higher catecholamine-activated lipolysis of the periovarian adipose tissue of 15-month-old *Ptn*^−/−^ mice than in *Ptn^+/+^* mice, leading to increasing flux of fatty acids into the circulation that may increase the concentrations in the local islet environment and stimulate insulin secretion [14,46].

Overall, our study provides valuable insights into the role of PTN in the maintenance of the morphology and functionality of endocrine pancreas in later life. Constitutive pleiotrophin deficiency contributes to morphological changes in pancreatic islets, accelerating age-related events such as glucose intolerance and decreased insulin sensitivity. The progressive decline in islet size, the disrupted insulin and glucagon ratio, and the alterations in insulin secretion mechanisms further highlight the critical role of PTN in maintaining pancreatic homeostasis during aging. Although the present study was only performed in female mice, unpublished results for the group indicate that the changes observed in the pancreas due to constitutive deletion of pleiotrophin were similar in males and females.

## 4. Materials and Methods

### 4.1. Animals

PTN genetically deficient (*Ptn*^−/−^*)* mice were generated as previously described [47,48]. Female knock-out (*Ptn*^−/−^) and wild-type (*Ptn*^+/+^) C57BL/6J mice were housed at 22–24 °C with 12-h/12-h light–dark cycles, from 08:00 to 20:00 h, with free access to water and chow diet (Panlab, Barcelona, Spain).

Animals were handled and maintained in accordance with the European Union Laboratory Animal Care Rules (Directive 2010/63/EU of the European Parliament and of the Council on the protection of animals used for scientific purposes) and protocols were approved by the Animal Research Committee of CEU San Pablo University and by Comunidad de Madrid (PROEX 137/18). Mice of 9 and 15 months of age from each genotype were fasted for 6 h and killed by decapitation. Plasma was collected and stored at −80 °C, and pancreata were dissected and fixed in 4% paraformaldehyde or used for islet isolation.

### 4.2. Reagents

The general reagents citrate, HBSS (Hanks’ Balanced Salt Solution), NaHCO_3_, HEPES, RPMI 1640, and penicillin–streptomycin were obtained from Sigma-Aldrich (Madrid, Spain) and BSA from PanReac AppliChem (Madrid, Spain).

### 4.3. Plasma Analysis and Glucose Tolerance Tests (IPGTT)

Glucose was determined via enzymatic tests (GOD-PAP Roche Diagnostics, Germany) in 3, 6, 9, 12, and 15-month-old *Ptn*^−/−^ and wild-type mice. Plasma insulin was determined using immunoassay kits (10-1247-10 Mercodia, Uppsala, Sweden). Plasma glucagon and GIP (glucose-dependent insulinotropic polypeptide) were measured with a Bioplex pro-Mouse diabetes Immunoassay kit (Bio-Rad, Hercules, CA, USA). IPGTT were performed in 6 h-fasted randomly selected mice; blood glucose was measured with a glucometer at 0, 10, 15, 20, 30, 45, and 60 min after intraperitoneal administration of glucose (2 g/kg) and the AUCs for glucose were calculated. HOMA-IR was calculated using fasting glucose and insulin as previously validated and described [49].

### 4.4. Histological Studies and Immunofluorescence

Formalin-fixed paraffin-embedded pancreas sections (3 µm) of 9- and 15-month-old *Ptn*^−/−^ and wild-type mice were stained with hematoxylin–eosin and analyzed in an Aperio Versa 8 (Leica Microsystems, Mannheim, Germany) optical microscope with the observer being blind to tissue identity using the Aperio ImageScope version 12.4.0.5043 software from Leica. We classified pancreatic islets according to major diameter into small (<150 µm), medium (150–300 µm), and big (>300 µm) islets, as described [50]. For immunofluorescence analysis, pancreatic slices were first processed using a deparaffination protocol. For the detection of certain proteins (GLUT2) antigen retrieval was performed. The samples were heated (95–100 °C) in citrate buffer pH 6 and rehydrated using PBS. To minimize the unspecific binding, blocking was performed using 1% BSA for 10 min. The pancreatic sections were incubated at 4 °C overnight with the corresponding primary antibodies (Table 1) diluted in Antibody Diluent (S0809 Dako, Glostrup, Hovedstaden, Denmark). The samples were washed twice with PBS and incubated for 60 min at room temperature with the corresponding secondary antibodies (Table 2) and 1:1000 Evans Blue (75491 bioMérieux, Rhone-Alpes, France) diluted in the antibody diluent. Further washes with PBS were carried out. Fluorescent mounting medium (S3023 Dako, Glostrup, Hovedstaden, Denmark) was used to preserve the fluorescence of the samples.

### 4.5. Image Acquisition and Analysis

Imaging was performed with a Leica STELLARIS 5 LIAchroic (Leica Microsystems, Mannheim, Germany) inverted confocal microscope, recording Alexa 488 (green) and Alexa 555 (red) fluorescence through separate channels. Confocal image stacks (10–50 images) from 4 pancreatic islets per animal were recorded from triple-stained sections, at 0.35 mm intervals with a 40× oil immersion lens (NA, 1.3, refraction index, 1.518 at zoom 1.0). Image analysis was performed with the observer being blind to tissue identity, using ImageJ version 1.54 software.

### 4.6. Islets Isolation

For pancreatic islets isolation, pancreata from both 9-month and 15-month-old *Ptn*^−/−^ and *Ptn^+/^^+^* mice were microinjected and digested with a 5 mL of 0.80 mg/mL collagenase P solution (REF, 11213865001 Roche, Germany) in HBSS and supplemented with 0.35 g/L of NaHCO_3_, 5.9575 g/L of HEPES and 2.5 g/L of BSA. Then, the pancreata were digested at 37 °C for 12 min with gentle agitation, and rapidly put on ice; collagenase action was stopped by adding 40 mL of cold-supplemented HBSS. For further tissue disruption, the pancreata were passed through 14G needles. Islets were hand-picked and cultured in 4 mL RPMI 1640 supplemented with 10% FBS (Biowest, Riverside, Newry and Mourne, UK) and 1% penicillin/streptomycin at 37 °C overnight.

### 4.7. Insulin Secretion Measurements

To measure insulin secretion, 2 sets of 10 size-matched isolated islets were placed in 1.5 mL tubes and incubated for 30 min with 1 mL of KRB + 0.1% BSA supplemented with 2.5 mM glucose (low glucose) or with 25 mM glucose (high glucose). After that, 600 µL of the media were centrifuged at 10,000 g for 1 min at 4 °C and the supernatants (secreted insulin) were stored at −80 °C. For total insulin islet content, at the end of the experiment, the islets underwent lysis with HCl:Ethanol (1:15 *v*/*v*), a −80 °C freeze-thaw cycle, and neutralization using a Tris Base (Millipore, Madrid, Spain) (1.5 mM final concentration). Secreted insulin and total insulin content in the islets were determined using a specific mouse ELISA kit (as indicated above).

### 4.8. INS1E Cell Culture and In Vitro GSIS

The rat insulinoma cells, INS1E, were cultured in RMPI 1640 medium (Sigma, Madrid, Spain) supplemented with 10 mM HEPES, 2 g/L sodium bicarbonate, 1 mM sodium pyruvate, 2 mM L-glutamine, 11.2 mM D-glucose, 50 μM beta-mercaptoethanol, 100 U/mL penicillin–streptomycin, and 10% heat-inactivated fetal bovine serum (FBS) at 37 °C and 5% CO_2_. When the confluency reached 80%, cells were passed.

Cells were plated in 6-well plates (5 × 10^5^ cells/well) and were cultured in complete RMPI 1640 medium with 10% FBS at 37 °C and 5% CO_2_ for 24 h. Then cells were fasted for 24 h in complete RMPI 1640 medium with 3% FBS at 37 °C and 5% CO_2_. For the glucose-stimulated insulin secretion study, INS1E cells were incubated with glucose-free Kreb’s buffer solution (KRB; 119 mM NaCl, 4.7 mM KCl, 25 mM NaHCO_3_, 2.5 mM CaCl_2_, 1.2 mM MgSO_4_, 1.2 mM KH_2_PO_4_, 1% BSA, 10 mM Hepes) for 2 h at 37 °C. Subsequently, cells were incubated in KRB with or without recombinant mouse PTN (0.1 or 1.0 µg/mL, (Bio-Techne R&D Systems, Madrid, Spain)) and in the presence of 2.0 mM (LG) or 20 mM (HG) glucose for 60 min at 37 °C. Supernatants were collected and secreted insulin was determined using a specific rat ELISA kit.

### 4.9. Statistical Analysis

Results are expressed as mean ± SEM. If the data were not normally distributed, the statistical analyses were performed with the log-transformed values. Grubbs’ test was used to detect outliers. Student’s t-test was used for the statistical comparisons between two groups and Chi-square analysis was used to compare proportions of different islet sizes (small, medium, big) or the increase in glucose-stimulated insulin secretion. Comparisons between three or more groups were made via one- or two-way ANOVA, followed by a Student–Newman–Keuls post hoc test. These analyses were performed using GraphPad Prism (v.9). The Mann–Whitney U test was used to evaluate differences in the number–size distribution among age in groups from the same genotype, using RStudio version 4.3.2 software.

## 5. Conclusions

This is the first study demonstrating that pleiotrophin expression is essential to preserve the appropriate basal metabolism and proper islet functionality. Our results suggest that PTN plays an essential role in the maintenance of pancreatic structural integrity, islet morphology and function, and in the preservation of glucose tolerance throughout the aging process. Although further studies are needed to completely elucidate the specific molecular pathways and regulatory mechanisms involving pleiotrophin in metabolic diseases associated with altered glucose–insulin homeostasis, these findings enhance our understanding of the molecular mechanisms underlying metabolic disorders and emphasize the role of pleiotrophin as a crucial target in the endocrine pancreas for future research and therapeutic interventions.

## Figures and Tables

**Table 1:** Primary antibodies used for immunofluorescence analysis.

Primary Antibody	Species	Type	IF Dilution	Antigen Retrieval	Reference	Manufacturer
Insulin (2D11-H5)	Mouse	Monoclonal	1:800	No	sc-(8033)	Santa Cruz Biotechnology (Dallas, TX, USA)
Glucagon	Rabbit	Polyclonal	1:800	No	SAB4501137	Sigma-Aldrich (Madrid, Spain)
Somatostatin	Rabbit	Polyclonal	1:100	No	ES3479	ELK Biotechnology (Denver, CO, USA)
SNAP25 (EPR3275)	Rabbit	Monoclonal	1:250	No	ab109105	Abcam (Amsterdam, The Netherlands)
VAMP2 (EPR12790)	Rabbit	Monoclonal	1:500	No	ab181869	Abcam (Amsterdam, The Netherlands)
GLUT2	Rabbit	Polyclonal	1:500	Yes	07-1402	Millipore (Madrid, Spain)

**Table 2:** Secondary antibodies used for immunofluorescence analysis.

Secondary Antibody	Species	IF Dilution	Reference	Manufacturer
anti-Mouse IgG (H + L) Cross-Adsorbed Secondary Antibody, Alexa Fluor™ 488	Goat	1:1000	A11001	Invitrogen, ThermoFisher Scientific (Madrid, Spain)
anti-Rabbit IgG (H + L) Cross-Adsorbed Secondary Antibody, Alexa Fluor™ 555	Goat	1:1000	A21428	Invitrogen, ThermoFisher Scientific (Madrid, Spain)

## Data Availability

The original contributions presented in the study are included in the article/Supplementary Material. Data associated with this study are available upon reasonable request. Further inquiries can be directed to the corresponding author.

## Supplementary Materials

The following supporting information can be downloaded at: https://www.mdpi.com/article/10.3390/ijms252010960/s1.
