# Supplementary material for: Constitutive Pleiotrophin Deletion Results in a Phenotype with an Altered Pancreatic Morphology and Function in Old Mice
Source: Int J Mol Sci. 2024 Oct 11;25(20):10960. doi: 10.3390/ijms252010960 (PMC11507919; doi:10.3390/ijms252010960)
Supplement: Supplementary file 1 [file ijms-25-10960-s001.zip › ijms-3237671-supplementary.pdf]

Supplementary Tables and Figures

Supplementary Table S1. Chi-square analysis comparing Small vs Medium-Big sized pancreatic islets among the different experimental groups (9 months *Ptn*$^{+/+}$, 9 months *Ptn*$^{-/-}$, 15 months *Ptn*$^{+/+}$, 15 months *Ptn*$^{-/-}$). Differences between experimental groups are shown by: *: $p < 0.05$; **: $p < 0.01$.

Small vs Medium-Big		
9 months *Ptn*$^{+/+}$ vs 9 months *Ptn*$^{-/-}$	n.s	p=0.2424
9 months *Ptn*$^{+/+}$ vs 15 months *Ptn*$^{+/+}$	*	p=0.0245
15 months *Ptn*$^{+/+}$ vs 15 months *Ptn*$^{-/-}$	**	p=0.0077
9 months *Ptn*$^{-/-}$ vs 15 months *Ptn*$^{-/-}$	n.s	p=0.1112

Supplementary Table S2. Chi-square analysis comparing Big vs Small-Medium sized pancreatic islets among the different experimental groups (9 months *Ptn*$^{+/+}$, 9 months *Ptn*$^{-/-}$, 15 months *Ptn*$^{+/+}$, 15 months *Ptn*$^{-/-}$). Differences between experimental groups are shown by: *: $p < 0.05$; ***: $p < 0.001$.

Big vs Small-Medium		
9 months *Ptn*$^{+/+}$ vs 9 months *Ptn*$^{-/-}$	n.s	*p*=0.1351
9 months *Ptn*$^{+/+}$ vs 15 months *Ptn*$^{+/+}$	n.s	*p*=0.6006
15 months *Ptn*$^{+/+}$ vs 15 months *Ptn*$^{-/-}$	*	*p*=0.0113
9 months *Ptn*$^{-/-}$ vs 15 months *Ptn*$^{-/-}$	***	*p*=0.0006

Supplementary Table S3. Increment of insulin secretion by isolated islets in the presence of high versus low glucose concentrations (GSIS) from animals of the same age and genotype. Data are expressed as fold change versus low glucose.

Fold change in glucose-stimulated insulin secretion	
9 months *Ptn*$^{+/+}$ High Glucose vs 9 months *Ptn*$^{+/+}$ Low Glucose	4.41
9 months *Ptn*$^{-/-}$ High Glucose vs 9 months *Ptn*$^{-/-}$ Low Glucose	1.53
15 months *Ptn*$^{+/+}$ High Glucose vs 15 months *Ptn*$^{+/+}$ Low Glucose	2.26
15 months *Ptn*$^{-/-}$ High Glucose vs 15 months *Ptn*$^{-/-}$ Low Glucose	1.62

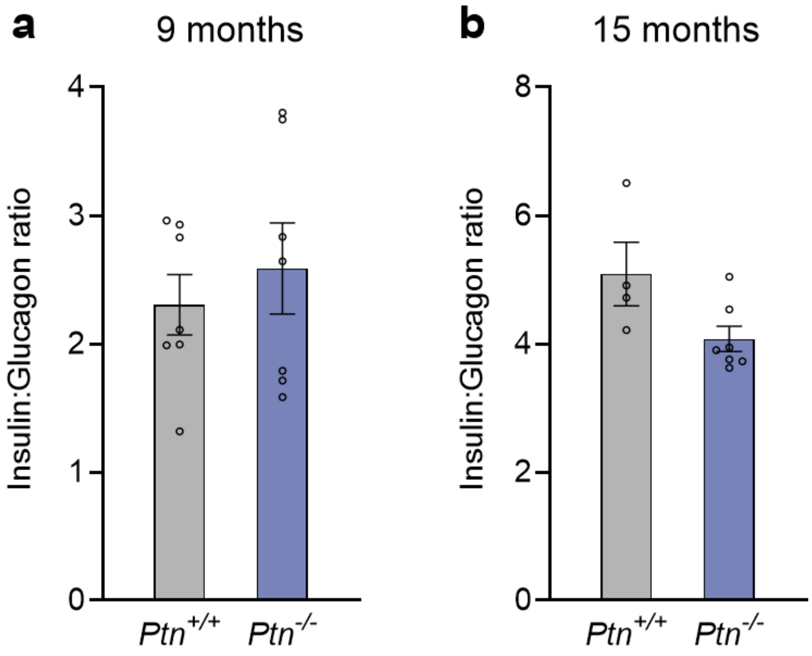

Supplementary Figure S1. Insulin:glucagon ratio from the fluorescence intensity quantification of 9- and 15-month-old *Ptn*$^{+/+}$ and *Ptn*$^{-/-}$ mice.
